# S177. FRONTAL CORTICAL PLASTICITY IN SCHIZOPHRENIA PATIENTS EXAMINED BY LTP-INDUCING ANODAL TDCS AND REPETITIVE EEG

**DOI:** 10.1093/schbul/sby018.964

**Published:** 2018-04-01

**Authors:** Benjamin Pross, Melina Siamouli, Oliver Pogarell, Peter Falkai, Alkomiet Hasan, Wolfgang Strube

**Affiliations:** Ludwig Maximilians University Munich

## Abstract

**Background:**

Frontal cortical deficits have repeatedly been shown to be relevant in the development of psychiatric disorders and are supposed to evoke characteristic psychiatric and cognitive symptoms in schizophrenia. It is assumed that plasticity and connectivity impairments following non-invasive brain stimulation, which are observed as common patterns in the motor system of schizophrenic patients, are as well present in frontal cortical areas and cause the mentioned dysfunctions. Until now experimental evidence is lacking substantiating that this hypothesis is correct and both cortical regions show similar patterns of deficits. Hence, this study aimed to assess the plasticity and connectivity in the frontal cortex of schizophrenia patients.

**Methods:**

We applied anodal transcranial direct current stimulation (a-tDCS) to evoke long-term potentiation (LTP)-like plasticity in the dorsolateral prefrontal cortex (DLPFC). This non-invasive brain stimulation has been demonstrated to evoke plasticity in frontal cortical regions. As tDCS modulates cortical activity we employed electroencephalography (EEG) measurements to trace potential deficits in patients with schizophrenia compared to healthy participants. In total 20 schizophrenia patients and 20 age, gender and handedness matched healthy controls received 13Min of a-tDCS (1mA). EEG was measured before and after plasticity induction (up to 50 minutes) to record neuronal changes in excitability and plasticity.

**Results:**

First analyses obtained a significant EEG alpha-activity change after LTP application in the frontal cortex of schizophrenia patients. This effect remained stable up to 50 minutes following a-tDCS stimulation.

**Discussion:**

We were able to show for the first time that anodal tDCS is capable of inducing stable EEG alpha-activity changes in the frontal cortex of schizophrenia patients. Future analyses will focus on differences to healthy participants, which we hypothesize to show similar but stronger patterns of activity changes after a-tDCS stimulation.

